# Fomentations

**Published:** 1873-04

**Authors:** 


### FOMENTATIONS.

This is a convenient and easily accessible means of relieving and curing a great variety of pains and ailments, and every mother and daughter should become thoroughly and practically acquainted with their proper mode of application, appropriate in all cases where there is no inflammation. Whether they should be cold or hot, depends on the sensation of the patient. That which is most agreeable is most beneficial.

Take a piece of woolen flannel of four, five, or six thicknesses ; put it in boiling water and wring it out with the hands, or enveloped in a towel ; lay this wrung-out flannel, which is now hot, smoothly over the ailing part, and cover that with another thicker flannel or blanket, so as to keep in all the steam ; in five minutes replace it with another, allowing as little cold air as possible to come to the body, especially to the fomented part ; these fomentations may be continued for fifteen or twenty minutes at a time ; particularly applicable in bruises, in cuts, when the skin is discolored, and in all dull pains.

When the parts are of a fiery, red color, and the pains are sharp, piercing, quick, throbbing, or darting, inflammation is indicated, and cold water should be used, not wringing the flannel dry, and renewed every five minntes.

